# Diseases of the Nervous System

**Published:** 1892-12

**Authors:** 


Diseases of the Nervous System. By J. A. Ormerod, M. D., Oxon. F. R. C. P., London. Medical Registrar and Demonstrator of Morbid Anatomy at St. Bartholomew's Hospital; Physician to the National Hospital for the Paralyzed and Epileptic, Queen's Square, and to the City of London Hospital for Diseases of the Chest, Victoria Park. With numerous illustrations. Philadelphia: P. Blakiston, Son & Co., 1012 Walnut street. 1892. Pp. 243; cloth, 8vo.

This is eminently a student's book, and is written evidently with the object of rendering the subject less appalling. He has done this by describing typical cases, and has not burdened the reader with too many variations from the typical forms. As the writer does not claim that the book is at all complete, or that it is anything more than an introduction to the larger and more complete treatises on the subject, it is not proper to criticise him for its incompleteness. We do believe, however, that it would have been better to be more definite in his etiology. For instance, he says of ant. polio-myelitis, its causes are practically unknown. He evidently ignores the fact that the disease presents a remarkable relation to season, as Sinkler showed that four-fifths of the cases commenced in the five hot months from May to September, and that Gowers showed that three-fourths of his seventy-one cases occurred from June to September. He omits all mention of the fact that exposure to cold has been, at times, an undoubted cause. Traumatism has also caused the disease. In his discussion of the treatment of this disease, he makes no mention of the treatment of the febrile stage, and in his treatment of the latter stage he does not mention strychnia. We believe that in his effort to be brief and simple in his style, he has omitted too much. What he has said he has said well and clearly, but not enough. J. W. P.

